# Development and validation of explainable machine learning models for predicting 3-month functional outcomes in acute ischemic stroke: a SHAP-based approach

**DOI:** 10.3389/fneur.2025.1678815

**Published:** 2025-12-02

**Authors:** Cheng-fang Chen, Zhan-yun Ren, Hui-hua Zong, Yi-tong Xiong, Yu Hong

**Affiliations:** 1 Department of Neurology, The Affiliated Yixing Hospital of Jiangsu University, Yixing, Jiangsu, China; 2 Information and Data Center, The Affiliated Yixing Hospital of Jiangsu University, Yixing, Jiangsu, China

**Keywords:** acute ischemic stroke, machine learning, functional outcome, SHAP, explainable artificial intelligence

## Abstract

**Objective:**

To develop and validate explainable machine learning models for predicting 3-month functional outcomes in acute ischemic stroke (AIS) patients using SHapley Additive exPlanations (SHAP) framework.

**Methods:**

This retrospective cohort study included 538 AIS patients admitted within 72 h of symptom onset. Patients were randomly divided into training (70%) and validation (30%) sets. Clinical, laboratory, and imaging data were collected. Least Absolute Shrinkage and Selection Operator regression was used for feature selection. Five machine learning models were developed: support vector machine, *k*-nearest neighbors, random forest, gradient boosting machine (GBM), and convolutional neural network. Model performance was evaluated using area under the receiver operating characteristic curve (AUC), accuracy, sensitivity, and specificity. SHAP analysis was applied to the best-performing model to enhance interpretability.

**Results:**

Among 538 patients (mean age 68.5 ± 12.7 years, 58.0% male), 34.2% had poor 3-month outcomes (mRS 3–6). The GBM achieved the best predictive performance with AUC of 0.91, accuracy of 0.81, sensitivity of 0.95, and specificity of 0.61 in validation set, significantly outperforming logistic regression (AUC = 0.78). The model demonstrated excellent calibration and superior net benefit in decision curve analysis across threshold probabilities of 0.1–0.7. SHAP analysis identified admission NIHSS score (30.8%), age (14.9%), and ASPECTS ≥7 (13.7%) as the most influential predictors, with neutrophil-to-lymphocyte ratio (10.1%) and platelet distribution width (9.7%) also contributing significantly to outcome prediction.

**Conclusion:**

Explainable machine learning models can accurately predict 3-month functional outcomes in AIS patients. The SHAP framework enhances model transparency, addressing interpretability barriers for clinical implementation while maintaining superior predictive performance.

## Introduction

Acute ischemic stroke (AIS) remains a leading cause of mortality and long-term disability worldwide, with approximately 13.7 million new cases annually (1). Despite advances in acute treatment strategies, including intravenous thrombolysis and endovascular thrombectomy, functional outcomes among stroke survivors exhibit substantial heterogeneity (2). Accurate prediction of functional outcomes at 3 months post-stroke is crucial for optimizing treatment decisions, resource allocation, patient counseling, and design of rehabilitation programs (3, 4).

Traditional prognostic tools for AIS rely primarily on clinical scoring systems such as the National Institutes of Health Stroke Scale (NIHSS) and the Alberta Stroke Program Early CT Score (ASPECTS) (5). While these tools provide valuable information, they often fail to capture complex interactions among multiple prognostic factors and may not account for individual patient variability (6). Moreover, these conventional models typically focus on a limited number of variables, potentially overlooking important predictors that might contribute to outcome prediction (7).

Machine learning (ML) techniques have emerged as promising approaches for developing prediction models in stroke care, capable of processing high-dimensional data and identifying complex patterns that may not be apparent through conventional statistical methods (8). Recent studies have demonstrated that ML algorithms can achieve superior predictive performance compared to traditional statistical models in various clinical scenarios, including stroke outcome prediction (9). The integration of clinical, laboratory, and imaging data through ML frameworks offers the potential for more comprehensive and accurate prognostication (10).

However, the widespread clinical adoption of ML models has been hindered by their “black box” nature, which limits interpretability and transparency in clinical decision-making (11, 12). Clinicians are understandably reluctant to rely on prediction models whose reasoning processes remain opaque, particularly in high-stakes medical decisions (13). This challenge has prompted increasing interest in explainable artificial intelligence (XAI) techniques that can elucidate the decision-making processes of complex ML algorithms while maintaining their predictive performance (14).

Among various XAI approaches, SHapley Additive exPlanations (SHAP) has gained prominence for its theoretically sound basis in cooperative game theory and its ability to provide both global and local interpretations of model predictions (15). SHAP values quantify the contribution of each feature to individual predictions, offering insights into which factors drive specific outcomes and how they interact (16). Recent work has demonstrated the utility of interpretable machine learning with SHAP analysis in predicting 30-day readmission after stroke, highlighting the value of transparent AI models in stroke care decision-making (17). Despite the potential of SHAP for enhancing ML model transparency in clinical applications, its systematic implementation in stroke outcome prediction models remains limited.

To address this gap, we aimed to develop and validate explainable ML models for predicting 3-month functional outcomes in AIS patients using the SHAP framework. By combining the predictive power of ML algorithms with the interpretability afforded by SHAP analysis, our study seeks to provide clinicians with a reliable and transparent decision support tool for stroke prognostication. Additionally, we aimed to identify key predictors of functional outcomes and elucidate their relative importance and interactions, thereby contributing to a deeper understanding of stroke recovery determinants. Our study makes three key contributions: (1) systematic comparison of multiple ML algorithms with conventional models for 3-month stroke outcome prediction; (2) comprehensive SHAP-based interpretability analysis providing both global feature importance and individual patient-level explanations; and (3) rigorous clinical utility evaluation through decision curve analysis.

## Methods

### Study design and participants

This single-center retrospective cohort study included patients with acute ischemic stroke admitted to the Department of neurology at Yixing People’s Hospital of Jiangsu University between January 1st and December 31st. The study protocol was approved by the institutional ethics committee. All patients provided informed consent.

Inclusion criteria were: (1) age 18–85 years; (2) diagnosis of acute ischemic stroke according to the Chinese guidelines for diagnosis and treatment of acute ischemic stroke; (3) admission within 72 h of symptom onset; (4) complete clinical information during hospitalization; and (5) complete 3-month follow-up records. Exclusion criteria were: (1) history of severe cognitive impairment or psychiatric disorders; (2) concomitant malignancy or severe systemic disease; (3) missing key clinical data exceeding 20%; and (4) loss to follow-up at 3 months.

### Data collection

Patient data were retrospectively collected from the hospital information system and medical record archives. Demographic data included age, gender, body mass index, and medical history (hypertension, diabetes, hyperlipidemia, coronary heart disease, atrial fibrillation, and prior stroke). Clinical data obtained at admission included time from symptom onset to hospital arrival, National Institutes of Health Stroke Scale (NIHSS) score, blood pressure, and heart rate.

Laboratory data collected within 24 h of admission included complete blood count, comprehensive metabolic panel, coagulation profile, inflammatory markers, lipid profile, and homocysteine. Derived indices such as platelet distribution width, neutrophil-to-lymphocyte ratio, and platelet-to-lymphocyte ratio were calculated.

Imaging data were obtained from head CT or MRI scans, including infarct location (anterior vs. posterior circulation), presence of large vessel occlusion, and Alberta Stroke Program Early CT Score (ASPECTS). Treatment information included administration of intravenous thrombolysis, endovascular therapy, and antiplatelet therapy.

### Outcome measures

The primary outcome was functional status at 3 months post-stroke, assessed using the modified Rankin scale (mRS). Good functional outcome was defined as mRS 0–2, and poor outcome as mRS 3–6. The 3-month follow-up data were collected from outpatient records, telephone follow-ups, or readmission records.

### Logistic regression model development

The dataset was randomly divided into a training set (70%) and a validation set (30%). Univariate logistic regression analysis was first performed to screen factors related to 3-month functional outcome (*p* < 0.05). Variables with statistical significance in the univariate analysis were then included in multivariate logistic regression analysis, using forward stepwise selection to identify independent predictors and construct a logistic regression prediction model. The Hosmer–Lemeshow test was used to assess model goodness-of-fit, and the Nagelkerke *R*^2^ value was calculated to evaluate the model’s explanatory power.

### Machine learning model development and feature selection

For machine learning model development, Least Absolute Shrinkage and Selection Operator (LASSO) regression was applied for feature selection, with the optimal regularization parameter *λ* determined through 5-fold cross-validation to select the most predictive features. LASSO was chosen over SHAP-based feature selection because LASSO performs feature selection before model training, reducing dimensionality and computational complexity, while SHAP analysis was reserved for post-hoc interpretation of the final model to ensure transparency and provide clinical insights into individual predictions. Based on the features selected by LASSO, five machine learning models were constructed: support vector machine (SVM), *k*-nearest neighbors (KNN), random forest (RF), gradient boosting machine (GBM), and convolutional neural network (CNN). Hyperparameter optimization was performed using grid search with 5-fold cross-validation in the training set.

### Model evaluation

The predictive performance of models was evaluated using multiple metrics: accuracy, area under the receiver operating characteristic curve (AUC), sensitivity, specificity, positive predictive value (PPV), negative predictive value (NPV), and *F*_1_-score. Calibration was assessed using calibration curves and the Hosmer–Lemeshow test. Clinical utility was evaluated using decision curve analysis (DCA) to compare the net benefit of different models across various threshold probabilities.

Model comparison was performed using the DeLong test to assess differences in AUC. The prediction probability distributions for patients with good and poor outcomes were visualized to assess the models’ discriminative ability.

### Model interpretability analysis

To enhance model interpretability, SHapley Additive exPlanations (SHAP) analysis was applied to the best-performing model. SHAP values were calculated to quantify the contribution of each feature to the prediction results. SHAP summary plots were generated to visualize the magnitude and direction of feature impact on model output. SHAP dependence plots were created to explore the relationship between feature values and their impact on predictions. SHAP waterfall plots for typical cases were used to illustrate individual feature contributions to specific predictions. SHAP cumulative effect plots were constructed to demonstrate the accumulation of feature impacts on prediction probabilities.

### Statistical analysis

Continuous variables were presented as mean ± standard deviation or median (interquartile range) as appropriate, and categorical variables as counts and percentages. Comparisons between groups were performed using independent *t*-tests or Mann–Whitney *U* tests for continuous variables and chi-square tests or Fisher’s exact tests for categorical variables.

Multivariate logistic regression was used to identify independent predictors of functional outcome, with odds ratios and 95% confidence intervals calculated. All statistical tests were two-sided, with *p* < 0.05 considered statistically significant.

Statistical analyses were performed using Python 3.9 (Python Software Foundation) for machine learning modeling and R 4.0 (R Foundation for Statistical Computing) for conventional statistical analyses. The SHAP library was used for model interpretation analyses.

## Results

### Baseline patient characteristics

This study included 632 patients with acute ischemic stroke, with 443 (70%) in the training set and 189 (30%) in the validation set. Ninety-four patients (14.9%) were excluded from the final analysis due to missing data exceeding our predefined threshold of 20% for key variables. The primary reasons for missing data included incomplete laboratory test results (*n* = 52, 55.3%), lack of complete imaging documentation (*n* = 28, 29.8%), and unavailable 3-month follow-up data (*n* = 14, 14.9%). Missing data analysis revealed no significant differences in baseline characteristics between included and excluded patients (all *p* > 0.05), suggesting that data were missing at random and unlikely to introduce systematic bias. The actual complete data analysis sample consisted of 538 cases (377 in the training set and 161 in the validation set). Among the 538 patients, 354 (65.8%) achieved good 3-month outcomes (mRS 0–2) and 184 (34.2%) had poor outcomes (mRS 3–6), representing a relatively balanced class distribution (approximately 2:1 ratio) favorable for machine learning model development without requiring resampling techniques.

As shown in Table 1, there were no statistically significant differences in baseline characteristics between the training and validation sets (*p* > 0.05), indicating balanced randomization. The mean age of patients was 68.5 ± 12.7 years, with males accounting for 58.0%. Supplementary Table 1 presents the comparison of baseline characteristics between outcome groups. Patients with poor outcomes were significantly older (72.1 ± 11.8 vs. 66.7 ± 12.8 years, *p* < 0.001), had higher admission NIHSS scores [median 14 (10–20) vs. 6 (3–9), *p* < 0.001], and more frequently had large vessel occlusion (48.9% vs. 28.5%, *p* < 0.001). Poor outcome patients also exhibited higher inflammatory markers including neutrophil-to-lymphocyte ratio [5.2 (3.4–8.1) vs. 3.1 (2.0–4.8), *p* < 0.001] and platelet distribution width (17.6 ± 2.5% vs. 16.4 ± 2.2%, *p* < 0.001), while having lower albumin levels (37.8 ± 5.1 vs. 39.5 ± 4.5 g/L, *p* < 0.001) and less favorable ASPECTS scores (68.5% vs. 83.3% with ASPECTS ≥7, *p* < 0.001).

**Table 1:** Comparison of baseline characteristics between training group, validation group and total sample.

Characteristics	Training group (*n* = 377)	Validation group (*n* = 161)	Total sample (*n* = 538)	*p*-value
Demographic characteristics				
Age (years, mean ± SD)	68.7 ± 12.5	67.9 ± 13.1	68.5 ± 12.7	0.542
Gender (male, *n*, %)	219 (58.1)	93 (57.8)	312 (58.0)	0.943
Body mass index (kg/m^2^, mean ± SD)	24.2 ± 3.7	23.9 ± 4.0	24.1 ± 3.8	0.395
Medical history (*n*, %)				
Hypertension	273 (72.4)	116 (72.0)	389 (72.3)	0.925
Diabetes	138 (36.6)	58 (36.0)	196 (36.4)	0.887
Hyperlipidemia	210 (55.7)	88 (54.7)	298 (55.4)	0.823
Coronary heart disease	87 (23.1)	37 (23.0)	124 (23.0)	0.981
Atrial fibrillation	62 (16.4)	27 (16.8)	89 (16.5)	0.919
Prior stroke	117 (31.0)	50 (31.1)	167 (31.0)	0.988
Clinical assessment				
Admission NIHSS score (median, IQR)	8 (4–14)	8 (4–15)	8 (4–14)	0.756
Onset to admission time (hours, median, IQR)	6.1 (2.7–18.2)	6.5 (3.0–19.1)	6.2 (2.8–18.5)	0.612
Systolic BP (mmHg, mean ± SD)	157.2 ± 28.1	155.9 ± 29.0	156.8 ± 28.4	0.624
Diastolic BP (mmHg, mean ± SD)	89.9 ± 16.0	89.3 ± 16.7	89.7 ± 16.2	0.708
Laboratory findings				
White blood cell count (×10^9^/L, median, IQR)	8.8 (7.0–11.1)	9.1 (7.3–11.4)	8.9 (7.1–11.2)	0.478
Platelet count (×10^9^/L, median, IQR)	199 (163–242)	196 (160–239)	198 (162–241)	0.734
Platelet distribution width (%, mean ± SD)	16.9 ± 2.3	16.6 ± 2.6	16.8 ± 2.4	0.213
Neutrophil/Lymphocyte ratio (median, IQR)	3.7 (2.3–6.1)	4.0 (2.5–6.4)	3.8 (2.4–6.2)	0.382
Platelet/Lymphocyte ratio (median, IQR)	141 (107–187)	144 (110–192)	142 (108–189)	0.567
Total bilirubin (μmol/L, median, IQR)	14.3 (10.9–19.8)	13.9 (10.6–19.2)	14.1 (10.8–19.6)	0.658
Albumin (g/L, mean ± SD)	39.0 ± 4.7	38.7 ± 5.0	38.9 ± 4.8	0.489
Creatinine (μmol/L, median, IQR)	77 (64–94)	79 (66–97)	78 (65–95)	0.456
Homocysteine (μmol/L, median, IQR)	14.9 (11.3–19.8)	14.6 (11.0–19.2)	14.8 (11.2–19.6)	0.672
Imaging features (*n*, %)				
Infarct location				0.785
Anterior circulation	275 (73.0)	117 (72.7)	392 (72.9)	
Posterior circulation	102 (27.0)	44 (27.3)	146 (27.1)	
Large vessel occlusion	132 (35.0)	57 (35.4)	189 (35.1)	0.930
ASPECT score ≥7	295 (78.2)	126 (78.3)	421 (78.3)	0.989
Treatment modalities (*n*, %)				
IV thrombolysis	164 (43.5)	70 (43.5)	234 (43.5)	0.997
Endovascular therapy	109 (28.9)	47 (29.2)	156 (29.0)	0.946
Antiplatelet therapy	349 (92.6)	149 (92.5)	498 (92.6)	0.978
Outcome				
3-Month poor outcome (mRS 3–6)	129 (34.2)	55 (34.2)	184 (34.2)	0.992

Continuous variables are presented as mean ± standard deviation or median (interquartile range); categorical variables are presented as count (percentage). Data were randomly divided into training (*n* = 377) and validation (*n* = 161) groups at a 7:3 ratio. The total sample column shows the combined statistics of both groups. IQR, interquartile range; NIHSS, National Institutes of Health Stroke Scale; ASPECT, Alberta Stroke Program Early CT Score; mRS, modified Rankin scale.

### Univariate and multivariate analysis of factors affecting functional outcome

Table 2 presents the results of univariate and multivariate analyses of factors affecting 3-month functional outcome. Univariate analysis showed that age, female gender, hypertension, diabetes, atrial fibrillation, history of previous stroke, admission NIHSS score, systolic blood pressure, white blood cell count, platelet distribution width, neutrophil/lymphocyte ratio, platelet/lymphocyte ratio, creatinine, homocysteine level, anterior circulation infarction, and large vessel occlusion were positively correlated with poor outcome (*p* < 0.05). Albumin level, ASPECT score ≥7, and intravenous thrombolysis were associated with good outcome (*p* < 0.001). Multivariate logistic regression analysis revealed that age (OR = 1.049, 95% CI: 1.024–1.075, *p* < 0.001), female gender (OR = 1.706, 95% CI: 1.052–2.768, *p* = 0.030), admission NIHSS score (OR = 1.265, 95% CI: 1.200–1.333, *p* < 0.001), white blood cell count (OR = 1.100, 95% CI: 1.015–1.192, *p* = 0.020), platelet distribution width (OR = 1.136, 95% CI: 1.015–1.272, *p* = 0.027), creatinine (OR = 1.071, 95% CI: 1.001–1.146, *p* = 0.046), and large vessel occlusion (OR = 2.214, 95% CI: 1.375–3.564, *p* = 0.001) were independent risk factors for poor outcome, while albumin level (OR = 0.931, 95% CI: 0.875–0.991, *p* = 0.025), ASPECT score ≥7 (OR = 0.398, 95% CI: 0.237–0.668, *p* < 0.001), and intravenous thrombolysis (OR = 0.561, 95% CI: 0.345–0.912, *p* = 0.020) were protective factors for good outcome. The multivariate model had a Nagelkerke *R*^2^ of 0.598, and the Hosmer–Lemeshow test indicated good model fit (*χ*^2^ = 6.83, *p* = 0.554). A logistic regression prediction model was constructed based on the multivariate analysis results in Table 2.

**Table 2:** Univariate and multivariate analysis of factors affecting 3-month functional outcome.

Variables	Univariate analysis			Multivariate analysis				
	OR	95% CI	*p*-value	*β* coefficient	Standard error	OR	95% CI	*p*-value
Age (per 1 year increase)	1.086	1.067–1.105	<0.001	0.048	0.013	1.049	1.024–1.075	<0.001
Gender (female vs. male)	1.532	1.062–2.211	0.022	0.534	0.247	1.706	1.052–2.768	0.030
Hypertension	1.732	1.142–2.630	0.010	—	—	—	—	—
Diabetes	1.466	1.014–2.119	0.042	—	—	—	—	—
Atrial fibrillation	2.146	1.365–3.376	0.001	—	—	—	—	—
Prior stroke	1.568	1.064–2.313	0.023	—	—	—	—	—
Admission NIHSS score (per 1 point increase)	1.312	1.261–1.365	<0.001	0.235	0.026	1.265	1.200–1.333	<0.001
Systolic BP (per 10 mmHg increase)	1.098	1.031–1.169	0.003	—	—	—	—	—
White blood cell count (per 1 × 10^9^/L increase)	1.124	1.045–1.209	0.002	0.095	0.041	1.100	1.015–1.192	0.020
Platelet distribution width (per 1% increase)	1.241	1.134–1.358	<0.001	0.128	0.058	1.136	1.015–1.272	0.027
Neutrophil/Lymphocyte ratio	1.286	1.189–1.390	<0.001	0.074	0.041	1.077	0.994–1.166	0.072
Platelet/Lymphocyte ratio	1.004	1.001–1.007	0.016	—	—	—	—	—
Albumin (per 1 g/L increase)	0.922	0.883–0.963	<0.001	−0.071	0.032	0.931	0.875–0.991	0.025
Creatinine (per 10 μmol/L increase)	1.087	1.011–1.169	0.023	0.069	0.034	1.071	1.001–1.146	0.046
Homocysteine (per 1 μmol/L increase)	1.049	1.019–1.080	0.001	—	—	—	—	—
Anterior circulation infarct	1.539	1.044–2.269	0.030	—	—	—	—	—
Large vessel occlusion	2.598	1.788–3.777	<0.001	0.795	0.243	2.214	1.375–3.564	0.001
ASPECT score ≥7	0.366	0.246–0.544	<0.001	−0.921	0.265	0.398	0.237–0.668	<0.001
IV thrombolysis	0.623	0.431–0.901	0.012	−0.578	0.248	0.561	0.345–0.912	0.020
Endovascular therapy	1.718	1.157–2.549	0.007	—	—	—	—	—

Multivariate model statistics: Constant: −0.156 (Standard error: 0.945); Model *χ*^2^: 289.4, *p* < 0.001; Hosmer–Lemeshow test: *χ*^2^ = 6.83, *p* = 0.554; Nagelkerke *R*^2^: 0.598.

OR, odds ratio; CI, confidence interval; *β*, regression coefficient. Multivariate analysis used binary logistic regression with forward stepwise method for variable selection.

### LASSO feature selection and importance ranking

**Figure 1:**
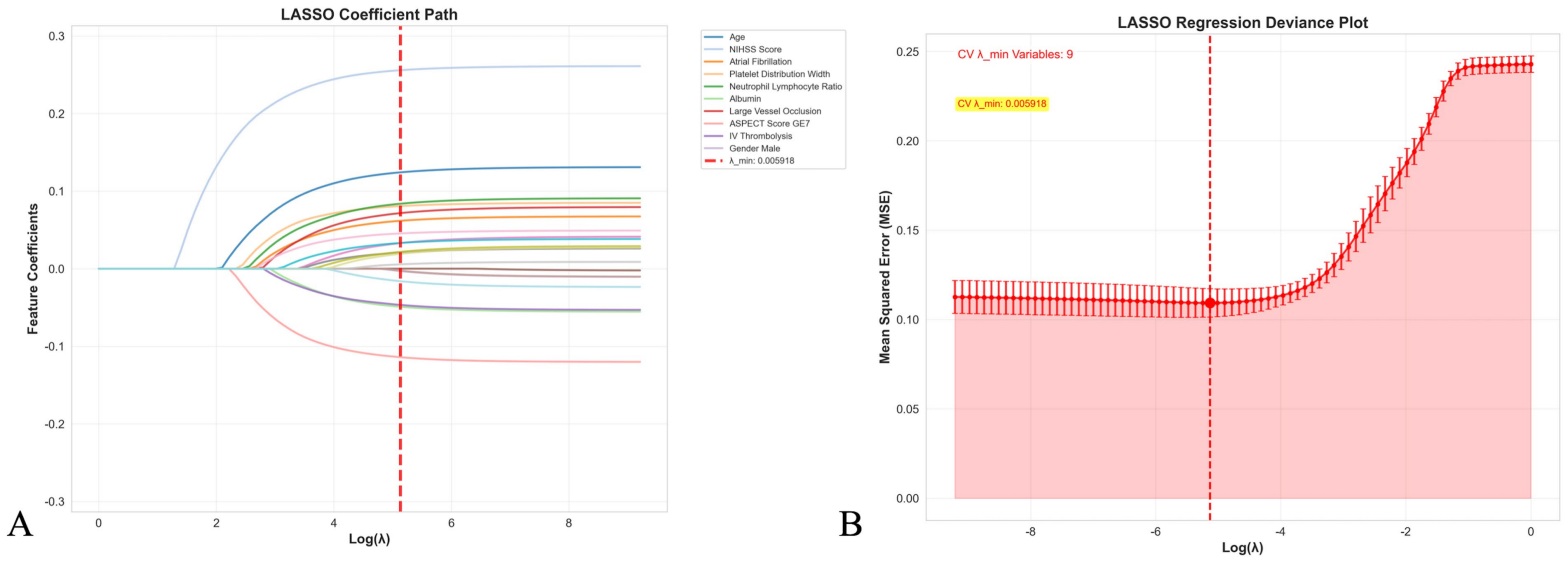
LASSO regression for feature selection in stroke outcome prediction. **(A)** LASSO coefficient path showing the evolution of feature coefficients as the regularization parameter *λ* increases. Each colored line represents a different feature, and the vertical red dashed line indicates the optimal *λ* value (0.005918) selected through cross-validation. **(B)** LASSO regression deviance plot displaying the mean squared error as a function of log(*λ*). The optimal *λ* value corresponds to the minimum cross-validation error, resulting in the selection of nine variables for subsequent machine learning model development. Error bars represent standard errors across cross-validation folds.

As shown in Figure 1, LASSO regression was used for feature selection and importance assessment. Figure 1A displays the LASSO coefficient path diagram, showing how variable coefficients gradually approach zero as the penalty parameter *λ* increases. Figure 1B presents the LASSO regression deviance plot, with the optimal *λ* value of 0.005918 determined through cross-validation, at which point nine variables were retained. These variables include admission NIHSS score, age, ASPECT score ≥7, neutrophil/lymphocyte ratio, platelet distribution width, large vessel occlusion, atrial fibrillation, albumin level, and intravenous thrombolysis, which were used for subsequent machine learning model construction.

### Comparison of machine learning model predictive performance

Based on the selected features, we constructed various machine learning models to predict 3-month functional outcome. As shown in Table 3, in the training set, the gradient boosting model performed best, with an accuracy of 0.81, AUC of 0.92, sensitivity of 0.98, and specificity of 0.56. The random forest model also performed well, with an AUC of 0.87 and accuracy of 0.78. In the validation set, the gradient boosting model likewise performed best, with an AUC of 0.91, accuracy of 0.81, sensitivity of 0.95, and specificity of 0.61. The random forest model ranked second in the validation set, with an AUC of 0.87, accuracy of 0.79, and specificity (0.74) higher than the gradient boosting model.

**Table 3:** Comparison of predictive performance across different machine learning models.

Model name	Accuracy	AUC	Sensitivity	Specificity	PPV	NPV	*F*_1_-score
Training set							
Gradient boosting	0.81	0.92	0.98	0.56	0.77	0.95	0.86
Random forest	0.78	0.87	0.83	0.71	0.81	0.74	0.82
CNN	0.80	0.86	0.91	0.65	0.79	0.83	0.85
Logistic regression	0.75	0.81	0.78	0.70	0.79	0.68	0.79
SVM	0.73	0.81	0.75	0.70	0.79	0.66	0.77
KNN	0.63	0.73	0.99	0.10	0.62	0.89	0.76
Validation set							
Gradient boosting	0.81	0.91	0.95	0.61	0.78	0.90	0.86
Random forest	0.79	0.87	0.83	0.74	0.83	0.74	0.83
KNN	0.75	0.83	0.66	0.89	0.90	0.64	0.76
CNN	0.74	0.81	0.79	0.66	0.78	0.69	0.78
SVM	0.71	0.78	0.75	0.65	0.76	0.64	0.76
Logistic regression	0.71	0.78	0.74	0.66	0.76	0.63	0.75

AUC, area under the curve, PPV, positive predictive value, NPV, negative predictive value, CNN, convolutional neural network, SVM, support vector machine, KNN, *k*-nearest neighbors.

**Figure 2:**
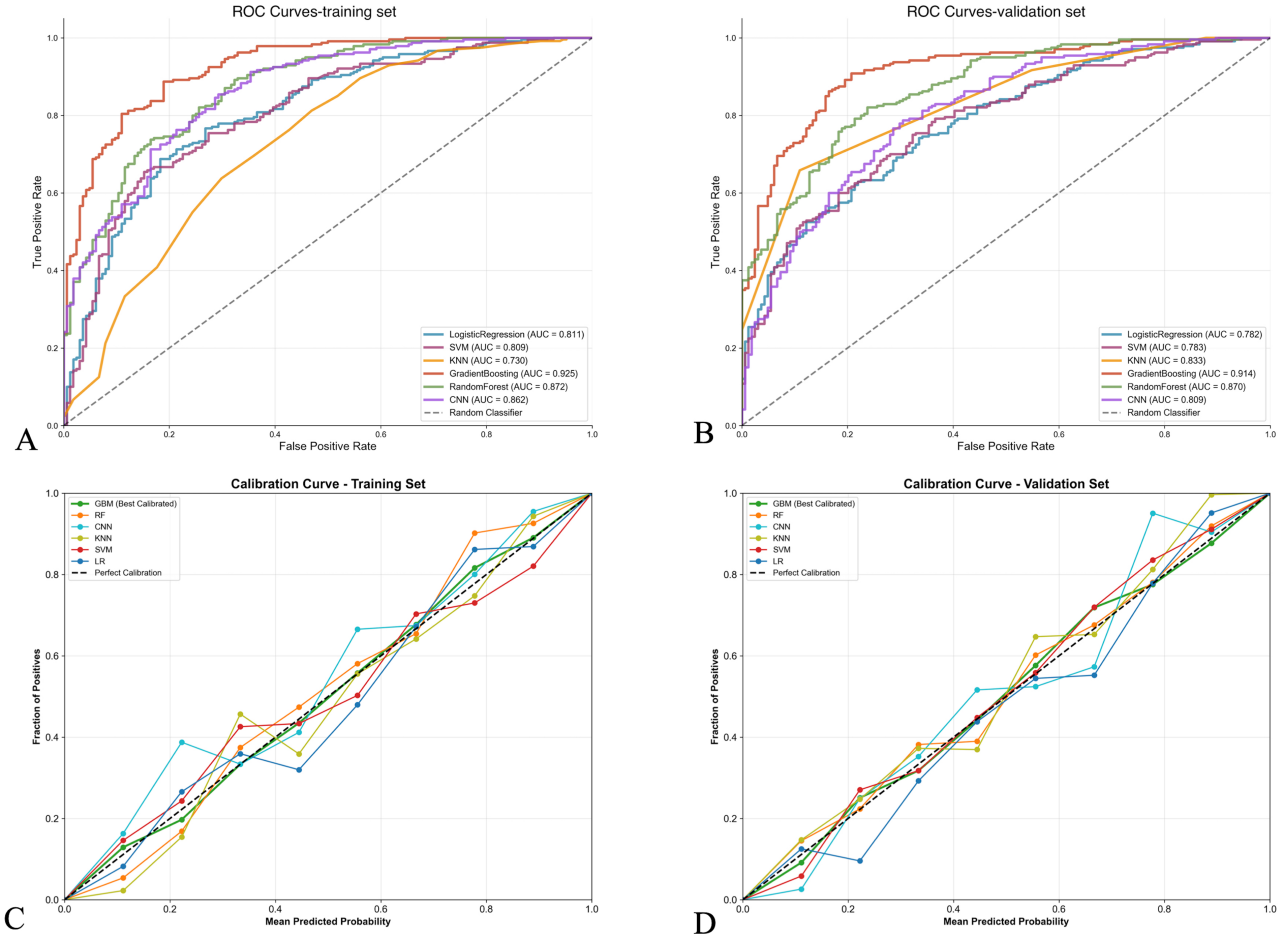
Performance comparison of machine learning models for stroke outcome prediction. **(A,B)** Receiver operating characteristic (ROC) curves for training and validation sets, respectively. The gradient boosting model achieved the highest area under the curve (AUC) in both sets (0.925 and 0.914, respectively), followed by random forest (0.872 and 0.870). **(C,D)** Calibration curves for training and validation sets, respectively. The diagonal dashed line represents perfect calibration. The gradient boosting model (red line) shows good calibration performance with predicted probabilities closely aligned with observed frequencies in both datasets.

Figure 2 further compares the ROC curves and calibration curves of various models in the training and validation sets. Figures 2A,B show that in both the training and validation sets, the ROC curve of the gradient boosting model is positioned highest, with AUCs of 0.925 and 0.914, respectively, followed by the random forest model (AUCs of 0.872 and 0.870, respectively). Figures 2C,D display the calibration curves, with the gradient boosting model (GBM) showing good calibration performance in both the training and validation sets, with predicted probabilities closely aligned with actual probabilities, approximating the ideal calibration line.

### Prediction probability distribution

**Figure 3:**
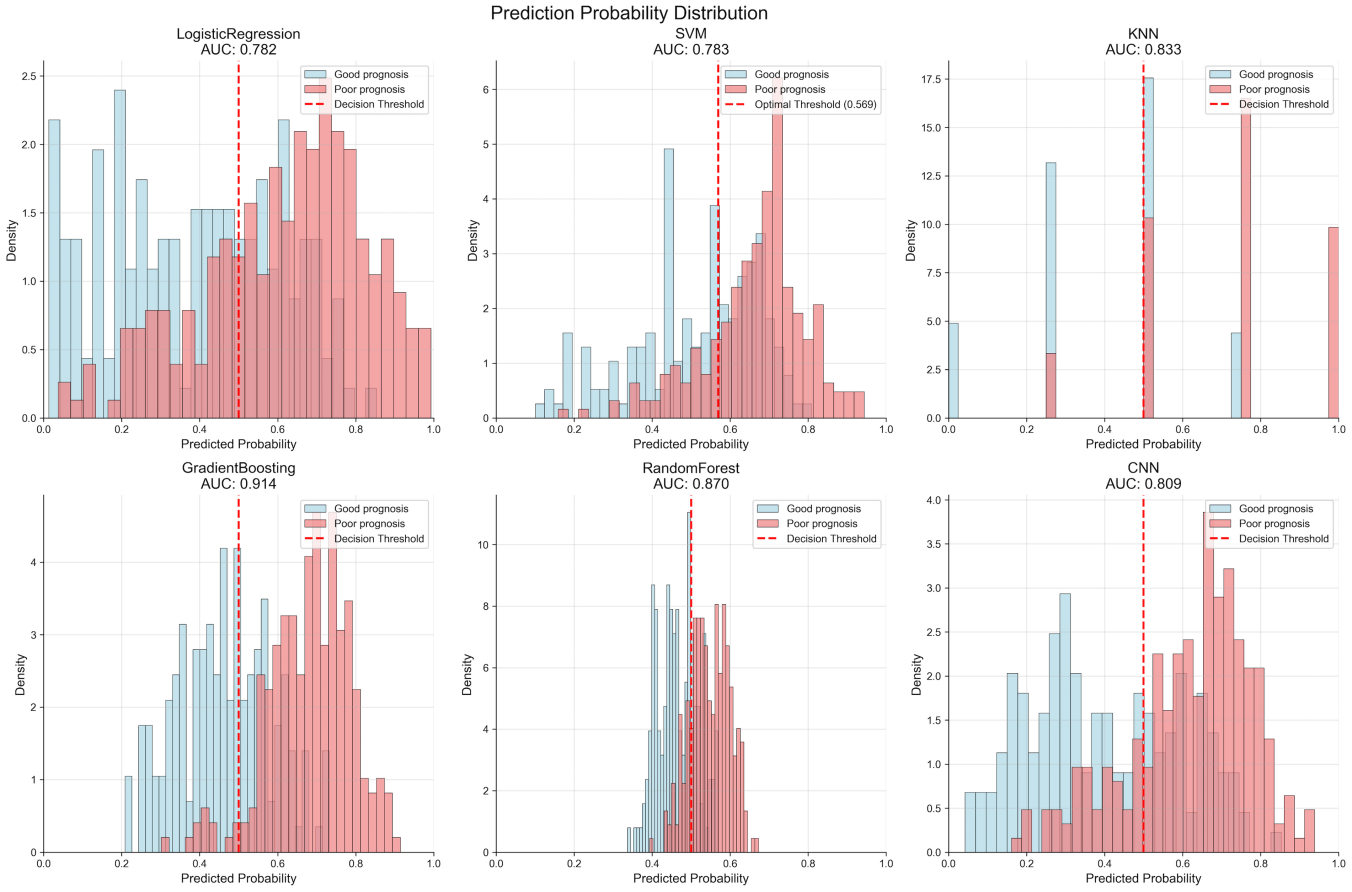
Prediction probability distributions of different machine learning models in the validation set. Each panel shows the distribution of predicted probabilities for patients with good prognosis (blue bars) and poor prognosis (red bars). The vertical dashed line indicates the optimal decision threshold for each model. The gradient boosting model (AUC = 0.914) demonstrates the best separation between the two outcome groups, with minimal overlap in probability distributions. Models are arranged by decreasing AUC performance: GradientBoosting > RandomForest > KNN > SVM > CNN > LogisticRegression.

Figure 3 shows the prediction probability distribution of various models in the validation set. The gradient boosting model (AUC of 0.914) demonstrates good classification ability, with clear separation between the prediction probability distributions of patients with good outcome (blue) and poor outcome (red). The random forest model (AUC of 0.870) also shows good classification performance, but the overlap area of prediction probabilities between the two groups is slightly larger than that of the gradient boosting model. The logistic regression model (AUC of 0.782) and SVM model (AUC of 0.783) have relatively weaker discriminative ability, with more overlap in prediction probability distributions between the two groups. The KNN model (AUC of 0.833) displays a unique distribution pattern, with prediction probabilities primarily concentrated at several discrete values, reflecting its classification characteristics based on neighboring samples.

### Decision curve analysis

**Figure 4:**
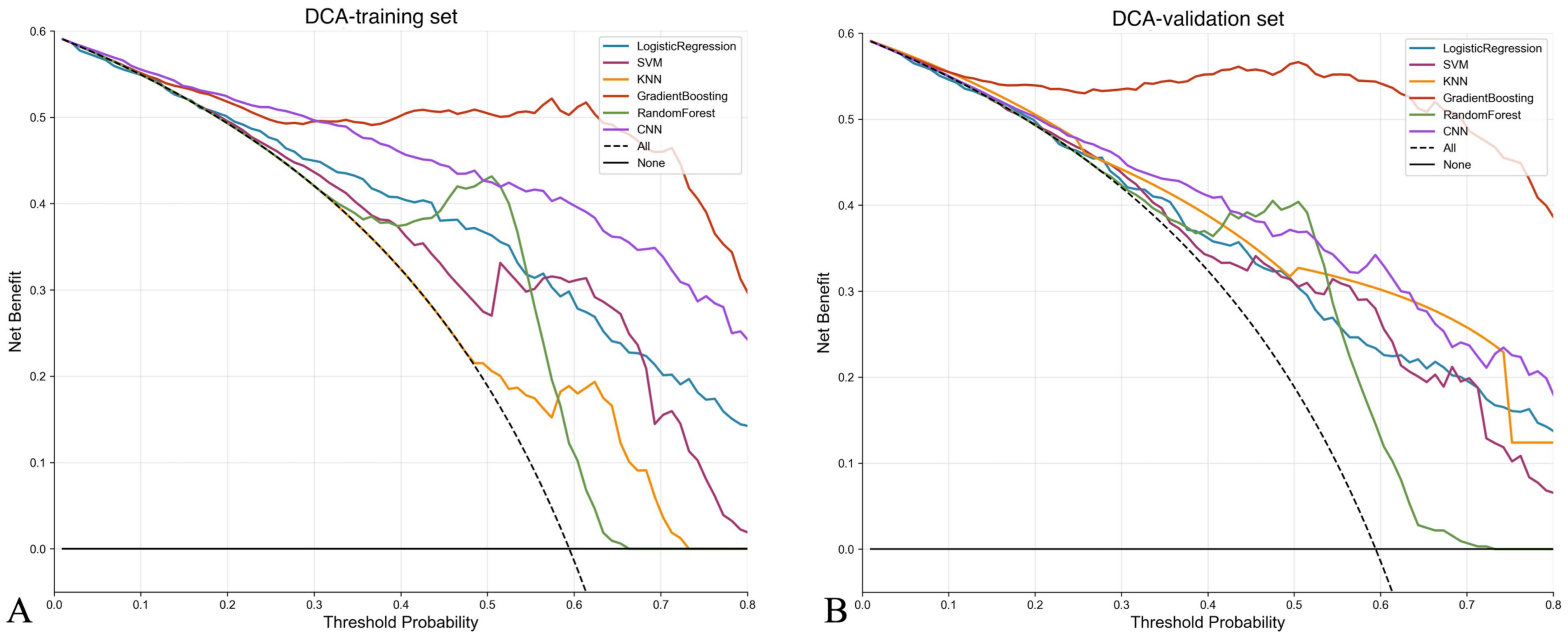
Decision curve analysis comparing the clinical utility of different prediction models. **(A,B)** Decision curves for training and validation sets, respectively. The *y*-axis represents net benefit, and the *x*-axis shows threshold probability. The “treat all” strategy assumes all patients receive intervention, while “treat none” assumes no patients receive intervention. All models demonstrate superior net benefit compared to these extreme strategies across most threshold probabilities. The gradient boosting model shows the highest net benefit in the clinically relevant threshold range (0.3–0.5), indicating superior clinical utility for decision-making.

Figure 4 presents the decision curve analysis results of different models. In both the training set (Figure 4A) and validation set (Figure 4B), all models show higher net benefit compared to “treat all” or “treat none” strategies. In the validation set, the gradient boosting model demonstrates the highest net benefit across most risk threshold ranges (0.1–0.7), with advantages particularly evident in the medium risk threshold range (0.3–0.5). This indicates that the model has high practical value in clinical decision support. Based on comprehensive evaluation results, the gradient boosting model (GBM) was selected as the final prediction model.

### SHAP analysis of GBM model

Table 4 lists the feature importance ranking of the GBM model. Admission NIHSS score ranks first (relative importance 30.8%), followed by age (14.9%) and ASPECT score ≥7 (13.7%). Other important features include neutrophil/lymphocyte ratio (10.1%), platelet distribution width (9.7%), large vessel occlusion (8.6%), atrial fibrillation (7.4%), albumin level (5.9%), and intravenous thrombolysis (5.6%).

**Table 4:** LASSO feature importance ranking.

Rank	Feature	Coefficient	Absolute value	Relative importance (%)
1	Admission NIHSS score	0.256	0.256	30.8
2	Age	0.124	0.124	14.9
3	ASPECT score ≥7	−0.114	0.114	13.7
4	Neutrophil/Lymphocyte ratio	0.084	0.084	10.1
5	Platelet distribution width	0.081	0.081	9.7
6	Large vessel occlusion	0.072	0.072	8.6
7	Atrial fibrillation	0.062	0.062	7.4
8	Albumin level	−0.049	0.049	5.9
9	IV thrombolysis	−0.047	0.047	5.6

LASSO, least Absolute Shrinkage and Selection Operator; importance score is the absolute value of LASSO regression coefficient; relative importance represents the percentage of each feature’s importance score relative to the total sum.

**Figure 5:**
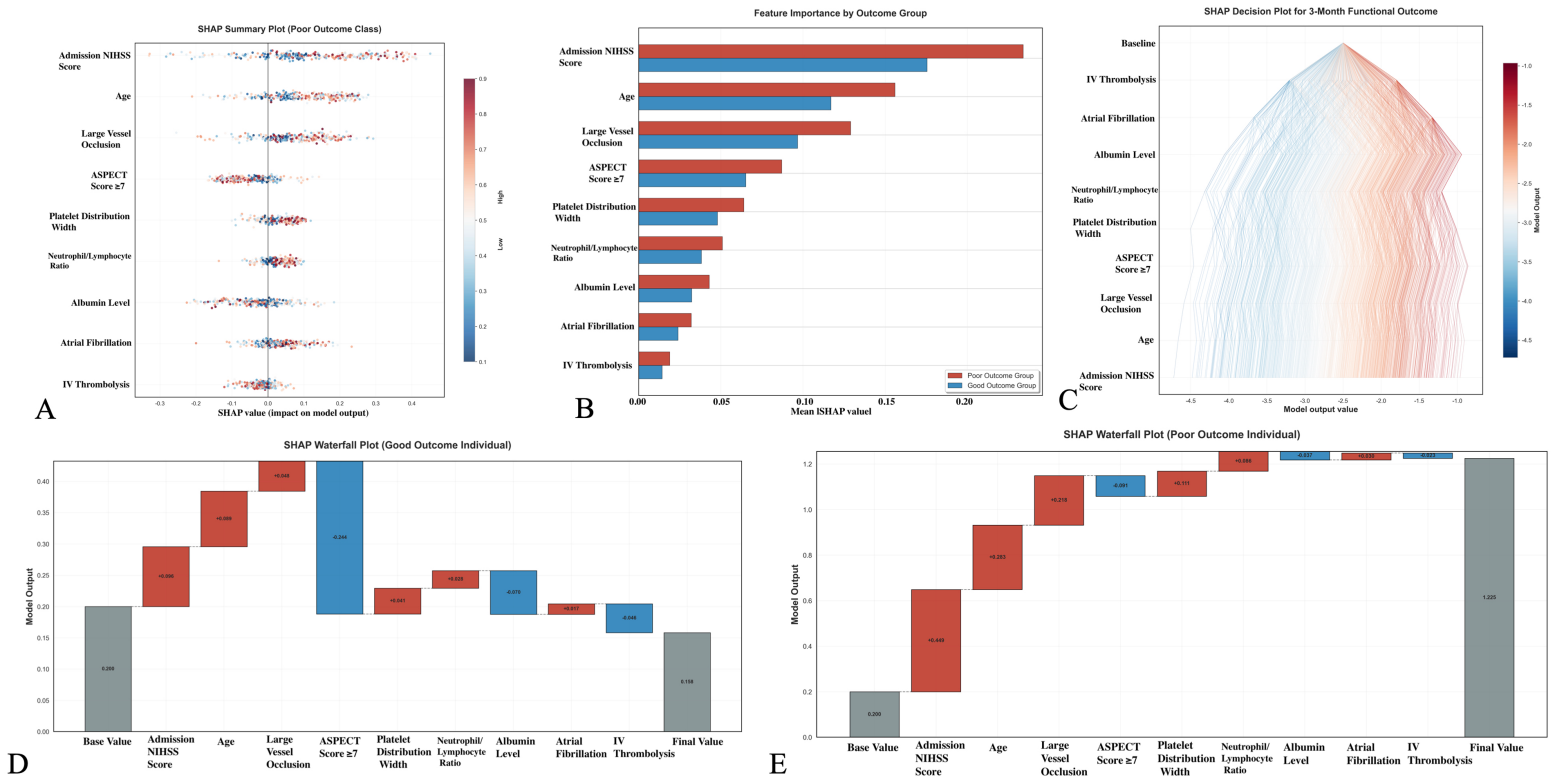
SHAP analysis of the gradient boosting model for stroke outcome prediction. **(A)** SHAP summary plot showing the impact of each feature on model predictions. Each dot represents a patient, with color indicating feature value (red = high, blue = low) and *x*-axis position showing SHAP value (impact on prediction). NIHSS score has the largest impact, followed by age and ASPECTS score. **(B)** Feature importance ranking by outcome group, comparing the relative importance of features between good and poor outcome patients. **(C)** SHAP decision plot showing the cumulative effect of features on prediction probability for 3-month functional outcome, with individual patient trajectories colored by risk level. **(D,E)** SHAP waterfall plots for representative cases showing individual feature contributions to specific predictions, illustrating how each variable pushes the prediction toward good or poor outcome.

Figure 5 shows the SHAP analysis results of the GBM model. Figure 5A is the SHAP summary plot, which visually displays the magnitude and direction of each feature’s contribution to model prediction, confirming that NIHSS score is the most critical factor affecting prognosis, followed by age and ASPECT score. Figure 5B is the SHAP dependence plot for NIHSS score, revealing the non-linear relationship between NIHSS score and prognosis, with accelerated contribution to poor outcome when NIHSS ≥10 points. Figure 5C is the SHAP waterfall plot for a typical case, showing the specific contribution values of various factors to individual prediction results. Figures 5D,E are SHAP cumulative effect plots, showing the cumulative impact process of features on prediction probabilities.

## Discussion

In this study, we developed and validated explainable machine learning models for predicting 3-month functional outcomes in patients with acute ischemic stroke. Our findings demonstrate that the gradient boosting model achieved the best predictive performance with an AUC of 0.91 in the validation set, outperforming conventional logistic regression (AUC 0.78) and other machine learning algorithms. Through SHAP analysis, we identified that admission NIHSS score, age, and ASPECT score were the most influential predictors of functional outcomes, accounting for approximately 59% of the total predictive contribution.

Our results align with recent investigations on machine learning applications in stroke outcome prediction. Wang et al. (13) conducted a systematic review of 70 studies developing machine learning models for stroke outcome prediction and found that advanced algorithms consistently outperformed conventional statistical models, with median AUCs ranging from 0.80 to 0.85. Similarly, Monteiro et al. (9) reported that ensemble learning methods, particularly gradient boosting, achieved superior performance (AUC 0.90) compared to logistic regression (AUC 0.85) in predicting functional independence after ischemic stroke, which corroborates our findings.

While the GBM model demonstrated excellent overall predictive performance (AUC 0.91), its specificity of 0.61 in the validation set indicates a relatively high false-positive rate, meaning the model may overpredict poor outcomes in some patients who actually achieve good functional recovery. This lower specificity could lead to potentially conservative treatment decisions or resource allocation for patients who might have favorable prognoses. This limitation reflects a trade-off inherent in our model optimization, which prioritized sensitivity (0.95) to minimize missing patients at high risk of poor outcomes, as failing to identify these high-risk patients may have more serious clinical consequences. In clinical practice, this model should be used as a complementary decision-support tool rather than a sole determinant, and clinicians should interpret predictions in conjunction with clinical judgment and individual patient circumstances.

The superior performance of the GBM model can be attributed to its ability to capture complex non-linear relationships and interactions among predictors. Beyond comparison with traditional logistic regression, our model’s performance should be contextualized against established clinical prognostic tools. The ASTRAL score (Acute Stroke Registry and Analysis of Lausanne) and THRIVE score (Totaled Health Risks in Vascular Events) are commonly used for stroke outcome prediction. Recent external validation studies reported AUCs of 0.77–0.85 for ASTRAL and 0.70–0.76 for THRIVE in predicting 3-month functional outcomes (18, 19). Our GBM model (AUC 0.91) demonstrates substantial improvement over these conventional scoring systems. This superior performance likely stems from our model’s ability to integrate broader clinical, laboratory, and imaging variables while capturing complex non-linear interactions that traditional additive scoring systems cannot accommodate. Previous study demonstrated that ensemble learning algorithms excel at modeling the multifaceted pathophysiological processes underlying stroke recovery, which often involve intricate interactions between demographic, clinical, and biological factors (20). Our model’s excellent calibration performance further supports its reliability for clinical application, as accurate probability estimation is crucial for risk stratification and decision-making.

Regarding predictor importance, our SHAP analysis revealed that admission NIHSS score was the most influential factor, which is consistent with previous studies. Boers et al. (21) found that initial stroke severity, as measured by the NIHSS, remained the strongest predictor of functional outcomes in their machine learning model. Similarly, van Os et al. (22) identified baseline NIHSS as the dominant predictor in their XGBoost model for 90-day mRS prediction. The non-linear relationship between NIHSS score and outcome probability observed in our SHAP dependence plot, with an accelerated contribution to poor outcome when NIHSS ≥10, provides clinically relevant insights for risk stratification.

Age emerged as the second most important predictor, consistent with findings from previous studies that advanced age independently predicted poor functional recovery through multiple pathophysiological mechanisms, including reduced neuroplasticity and higher comorbidity burden (23, 24). The ASPECT score, ranking third in our model, has been recognized as a critical imaging biomarker for outcome prediction. Guberina et al. (25) demonstrated that ASPECT scores effectively captured the extent of early ischemic changes and significantly influenced functional prognosis, supporting our findings.

Interestingly, our model identified several laboratory parameters as important predictors, including neutrophil/lymphocyte ratio and platelet distribution width. These inflammatory and hematological markers have gained increasing attention in stroke prognostication. Wu et al. (26) found that elevated neutrophil/lymphocyte ratio predicted poor functional outcomes after ischemic stroke, potentially reflecting the detrimental effects of neuroinflammation on recovery. Regarding platelet distribution width (PDW), our finding that it ranked fifth in importance (9.7%) provides novel insights into stroke prognostication. PDW reflects platelet size heterogeneity and activation status. Elevated PDW may indicate enhanced platelet activation and prothrombotic state, potentially contributing to microvascular dysfunction and impaired cerebral perfusion during the acute phase. Furthermore, platelet activation releases inflammatory mediators that exacerbate neuroinflammation and secondary brain injury. The inclusion of PDW in our model suggests that hematological markers beyond traditional complete blood count parameters may capture subtle pathophysiological processes affecting stroke recovery, warranting further investigation into platelet function biomarkers in personalized stroke management.

A notable strength of our study is the application of SHAP analysis to enhance model interpretability. Traditional machine learning models often function as “black boxes,” limiting their clinical adoption despite superior predictive performance. SHAP values provide a unified framework for explaining model predictions based on cooperative game theory, allowing for both global understanding of model behavior and local interpretation of individual predictions (27, 28). Our SHAP-based approach addresses the interpretability gap that clinical implementation of AI systems requires transparent decision-making processes to gain physicians’ trust and improve patient outcomes (29, 30).

The clinical utility of our model is further demonstrated by decision curve analysis, which showed higher net benefit compared to “treat all” or “treat none” strategies across a wide range of threshold probabilities. This aligns with findings from Hildesheim et al. (31), who advocated for decision curve analysis as an essential step in evaluating prognostic models’ clinical impact beyond traditional discrimination and calibration metrics. Our model’s superior net benefit in the medium risk threshold range (0.3–0.5) suggests particular value in clinical scenarios where decision uncertainty is highest.

From a clinical perspective, our findings have several important implications. First, the identification of key prognostic factors can guide resource allocation and early intervention strategies. Second, the quantification of each predictor’s contribution provides a framework for personalized risk assessment and targeted rehabilitation planning. Third, the model’s ability to identify high-risk patients may facilitate early aggressive management and specialized care pathways.

Despite these strengths, our study has several limitations. First, as a single-center retrospective study, our findings may be influenced by selection bias and institution-specific practices, potentially limiting generalizability. Our patient cohort was drawn from a tertiary hospital serving both urban and rural populations in Jiangsu Province, which may not fully represent stroke populations in other geographic regions or healthcare settings with different patient demographics, treatment protocols, or resource availability. To address these concerns, future studies should prioritize multicenter external validation across diverse healthcare systems and patient populations to establish the broader applicability and robustness of our predictive models. Second, while our sample size was adequate for model development, external validation in larger, multicenter cohorts is necessary to establish broader applicability. Third, our models primarily included clinical, laboratory, and basic imaging variables, without incorporating advanced imaging features such as perfusion parameters or detailed vessel characteristics, which might enhance predictive performance as demonstrated by Winzeck et al. (32).

Furthermore, we did not include genetic markers or emerging biomarkers of stroke recovery, which are increasingly recognized as important determinants of outcomes. Additionally, we focused on 3-month outcomes without examining longer-term functional trajectories, which might provide more comprehensive insights into recovery patterns.

Future research should address these limitations through prospective, multicenter validation studies with larger sample sizes and longer follow-up periods. Integration of advanced neuroimaging features, molecular biomarkers, and longitudinal assessment could further enhance predictive performance. Implementation studies evaluating the impact of model-guided decision-making on patient outcomes and resource utilization are also warranted to demonstrate real-world clinical benefits.

The integration of SHAP-based interpretability with high-performing gradient boosting represents a methodological advancement over previous stroke prediction studies. While earlier ML applications in stroke outcome prediction achieved competitive AUCs, they typically lacked comprehensive interpretability frameworks, limiting clinical trust and adoption. Our SHAP analysis not only quantifies global feature importance but also reveals non-linear relationships and provides patient-specific explanations through waterfall plots. This dual focus on accuracy and transparency addresses a critical barrier identified in recent systematic reviews of clinical AI implementation.

In conclusion, our study demonstrates that explainable machine learning models can accurately predict 3-month functional outcomes in acute ischemic stroke patients, with the gradient boosting algorithm showing superior performance. The SHAP analysis framework enhances model transparency by identifying key predictors and quantifying their contributions, addressing a critical barrier to clinical implementation. By combining predictive power with interpretability, our approach represents a promising step toward personalized stroke prognostication and precision medicine in acute stroke care.

## Data Availability

The raw data supporting the conclusions of this article will be made available by the authors, without undue reservation.
