# Supplementary material for: Development and validation of explainable machine learning models for predicting 3-month functional outcomes in acute ischemic stroke: a SHAP-based approach
Source: Front Neurol. 2025 Dec 2;16:1678815. doi: 10.3389/fneur.2025.1678815 (PMC12705366; doi:10.3389/fneur.2025.1678815)
Supplement: Supplementary file 1 [file Table_1.docx]

Supplementary Table 1: Comparison of Baseline Characteristics Between Good and Poor Outcome Groups

| Characteristics | Good Outcome (mRS 0-2) (n=354) | Poor Outcome (mRS 3-6) (n=184) | *P* Value |
| --- | --- | --- | --- |
| Demographic Characteristics |  |  |  |
| Age (years, mean±SD) | 66.7±12.8 | 72.1±11.8 | <0.001 |
| Gender (male, n, %) | 228 (64.4) | 84 (45.7) | <0.001 |
| Body Mass Index (kg/m², mean±SD) | 24.3±3.6 | 23.8±4.1 | 0.134 |
| Medical History (n, %) |  |  |  |
| Hypertension | 247 (69.8) | 142 (77.2) | 0.068 |
| Diabetes | 121 (34.2) | 75 (40.8) | 0.127 |
| Hyperlipidemia | 198 (55.9) | 100 (54.3) | 0.723 |
| Coronary Heart Disease | 78 (22.0) | 46 (25.0) | 0.436 |
| Atrial Fibrillation | 45 (12.7) | 44 (23.9) | 0.001 |
| Prior Stroke | 99 (28.0) | 68 (37.0) | 0.029 |
| Clinical Assessment |  |  |  |
| Admission NIHSS Score (median, IQR) | 6 (3-9) | 14 (10-20) | <0.001 |
| Onset to Admission Time (hours, median, IQR) | 6.0 (2.6-17.8) | 6.8 (3.2-20.1) | 0.189 |
| Systolic BP (mmHg, mean±SD) | 155.3±27.8 | 159.8±29.5 | 0.081 |
| Diastolic BP (mmHg, mean±SD) | 89.2±15.9 | 90.7±16.8 | 0.308 |
| Laboratory Findings |  |  |  |
| White Blood Cell Count (×10⁹/L, median, IQR) | 8.5 (6.8-10.4) | 9.8 (7.8-12.5) | <0.001 |
| Platelet Count (×10⁹/L, median, IQR) | 200 (165-243) | 195 (158-238) | 0.287 |
| Platelet Distribution Width (%, mean±SD) | 16.4±2.2 | 17.6±2.5 | <0.001 |
| Neutrophil/Lymphocyte Ratio (median, IQR) | 3.1 (2.0-4.8) | 5.2 (3.4-8.1) | <0.001 |
| Platelet/Lymphocyte Ratio (median, IQR) | 136 (104-179) | 153 (115-205) | 0.006 |
| Total Bilirubin (μmol/L, median, IQR) | 14.2 (10.9-19.5) | 13.9 (10.7-20.0) | 0.892 |
| Albumin (g/L, mean±SD) | 39.5±4.5 | 37.8±5.1 | <0.001 |
| Creatinine (μmol/L, median, IQR) | 76 (63-91) | 82 (68-101) | 0.003 |
| Homocysteine (μmol/L, median, IQR) | 14.2 (10.8-18.6) | 16.1 (12.3-21.5) | 0.001 |
| Imaging Features (n, %) |  |  |  |
| Infarct Location |  |  | 0.042 |
| Anterior Circulation | 251 (70.9) | 141 (76.6) |  |
| Posterior Circulation | 103 (29.1) | 43 (23.4) |  |
| Large Vessel Occlusion | 99 (28.0) | 90 (48.9) | <0.001 |
| ASPECTS ≥7 | 295 (83.3) | 126 (68.5) | <0.001 |
| Treatment Modalities (n, %) |  |  |  |
| IV Thrombolysis | 166 (46.9) | 68 (37.0) | 0.024 |
| Endovascular Therapy | 92 (26.0) | 64 (34.8) | 0.032 |
| Antiplatelet Therapy | 330 (93.2) | 168 (91.3) | 0.426 |

*Note: Continuous variables are presented as mean±standard deviation or median (interquartile range); categorical variables are presented as count (percentage). Good outcome was defined as modified Rankin Scale (mRS) 0-2 at 3 months; poor outcome as mRS 3-6. IQR: Interquartile Range; NIHSS: National Institutes of Health Stroke Scale; ASPECTS: Alberta Stroke Program Early CT Score.*
